# L‐Quebrachitol Enhances Sedative Effect of Diazepam Through GABAergic Pathway: Animal and Computational Studies

**DOI:** 10.1002/brb3.71185

**Published:** 2026-01-13

**Authors:** Asifa Asrafi, Mohammad Aslam, Md. Sakib Al Hasan, Mohammed Burhan Uddin, Emon Mia, Mohammad Y. Alshahrani, Sumaya Akter Bithi, Mst. Sumaia Akter, Md. Arif Hossain, Md. Torequl Islam

**Affiliations:** ^1^ Department of Biochemistry and Molecular Biology Gopalganj Science and Technology University Gopalganj Bangladesh; ^2^ Department of Pharmacy Gopalganj Science and Technology University Gopalganj Bangladesh; ^3^ Bioinformatics and Drug Innovation Laboratory BioLuster Research Center Ltd. Gopalganj Bangladesh; ^4^ Department of Chemistry, York College City University of New York Jamaica New York USA; ^5^ Central Labs King Khalid University Abha Saudi Arabia; ^6^ Department of Clinical Laboratory Sciences, College of Applied Medical Sciences King Khalid University Abha Saudi Arabia; ^7^ Pharmacy Discipline Khulna University Khulna Bangladesh

**Keywords:** animal study, GABA_A_ receptor, in silico studies, L‐quebrachitol, sedative effect

## Abstract

**Introduction:**

Insomnia and other sleep disorders are becoming increasingly prevalent worldwide, while current sedative medications such as benzodiazepines, though effective, are often limited by side effects and dependency risks. Therefore, identifying safe natural compounds with sedative potential is of growing scientific and clinical interest. L‐quebrachitol (LQL), a naturally occurring cyclitol compound with antioxidant, antimicrobial, and antidiabetic properties, has not been previously evaluated for its sedative effects. The aim of this study is to evaluate the potential sedative effects of LQL through both in vivo and in silico methods.

**Methods:**

In this experiment, 2‐day‐old broiler chicks (*Gallus gallus domesticus*) were given thiopental sodium (10 mg/kg, intraperitoneal [ip]) to induce sleep. LQL (1, 5, and 10 mg/kg, ip) and diazepam (2 mg/kg, ip) were administered alone or together to assess their synergistic or antagonistic effects on chicks. To assess its potential for interacting with the GABA_A_ receptor (α1 and β2 subunits), a molecular docking study was carried out.

**Result:**

According to the in vivo investigation, the results indicate that LQL decreased the latency period while extending the animal's sleep duration time in a dose‐dependent manner. Moreover, the combination of LQL‐10 (10 mg/kg) and diazepam 2 (2 mg/kg) showed (*p* < 0.05) enhanced sedative effects significantly by decreasing latency time and prolonging sleeping duration. In addition, LQL has a moderate binding affinity of −5.3 kcal/mol against the GABA_A_ receptor (α1 and β2 subunits), but forms strong hydrogen bond interactions and similar amino acid residues with standard drug diazepam, suggesting potential therapeutic effects. Further, LQL also demonstrated promising pharmacokinetic properties and low toxicity.

**Conclusion:**

These findings collectively enhance the potential of LQL as an effective sedative therapeutic agent. However, further research, including in vitro studies to confirm the molecular interactions and membrane permeability, followed by well‐designed clinical trials, is necessary to fully establish LQL as a safe and effective sedative agent.

## Graphical abstract

L‐quebrachitol (LQL) significantly enhanced the sedative effects of diazepam in thiopental sodium‐induced chicks by reducing sleep latency and prolonging duration. Molecular docking revealed LQL's interaction with GABA_A_ receptor (α1 and β2 subunits), supporting a GABAergic mechanism and highlighting its potential as a safe, natural sedative agent.

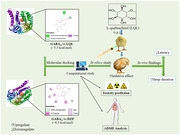

## 1 Introduction

Insomnia, which means difficulty with sleep duration, initiation, quality, or consolidation despite passable sleep opportunities, is an obvious problem in the present 24‐h society (Pagel et al. 2018; Bhuia et al. 2024). The most often occurring causes of insomnia are depression or anxiety, stress, a room that's cold or too hot, noise, uncomfortable beds, nicotine or caffeine and alcohol intake, illegal drugs including ecstasy or cocaine, and shift work (https://www.nhs.uk/conditions/insomnia/ accessed on January 22, 2025). Insomnia can occur over either a short time (acute) or a longer period (chronic) (Liu 2020). Up to 37% of people in the general population are thought to have acute symptoms of insomnia each year. About 10%–20% of people suffer from chronic insomnia, and those with lower socioeconomic levels, women, and older folks are more likely to have it (Ferini‐Strambi et al. 2021). Even yet, the majority of college students may experience inconsistent sleep patterns, which can lead to daily distress and functional impairment. People have been struggling with a range of sleep‐related problems in recent years as a result of the damaging effects of COVID‐19 throughout the state (Mukty et al. 2024). Insomnia can also result from a variety of other illnesses, including sexual dysfunction, somatic problems, Type 2 diabetes, hypertension, schizophrenia, and acute myocardial infarction (Pietikäinen et al. 2019).

Insomnia is highly prevalent and often coexists with other disorders; cognitive behavioral therapy for insomnia (CBT‐I) is the first‐line treatment, focusing on changing sleep patterns, controlling sleep‐wake cycles, and addressing negative thoughts about sleep (Morin and Buysse 2024). Again, reduced GABA activity can lead to heightened neuronal firing, contributing to symptoms such as restlessness, nervousness, and insomnia (Varinthra et al. 2024). Therefore, enhancing GABAergic transmission is a common therapeutic approach to calm the central nervous system (CNS) (Gulias‐Cañizo et al. 2021). To treat sleep disorders, current sleep aids such as melatonin agonists, non‐benzodiazepine hypnotics, and benzodiazepines are commonly utilized (McGowan et al. 2022; Reeve et al. 2017; Starownik et al. 2025; Sobolewski 2021). These treatments, although effective, long‐term insomnia treatments can cause side effects like poor coordination and fatigue (Ferdous et al. 2024). Again, according to Edinoff et al. (2021), benzodiazepine treatment is associated with several side effects, including drowsiness, dizziness, cognitive impairment, decreased concentration, muscle weakness, ataxia, dependence, tolerance, and withdrawal symptoms such as anxiety, insomnia, and irritability upon discontinuation (Edinoff et al. 2021), creating an urgent need for safer alternatives.

Natural products are bioactive compounds from natural sources that play a crucial role in drug discovery and disease management through their pharmacological properties (Aktar et al. 2024; Bithi et al. 2025). Natural sleep aids are becoming increasingly popular worldwide because of their effectiveness and safety (Shi et al., 2014; Aldhafiri et al. 2023). Phytochemicals like polyphenols, alkaloids, terpenes, and saponins contribute to the hypnotic effects of natural sleep remedies, with polyphenols acting through the GABA_A_ receptor (Cho and Shimizu 2015; Bruni et al. 2021; Montesano and Gallo 2021). A study by Sahin et al. (2024) demonstrated that a novel valerian extract significantly improved sleep quality and relaxation in mice by enhancing GABA and serotonin receptor activity, suggesting its strong sedative and anxiolytic potential (Sahin et al. 2024). Similarly, Pinheiro et al. (2016) demonstrated that Valeriana officinalis can be effectively used for conscious sedation, supporting its traditional use as a natural sleep inducer and in the management of insomnia (Pinheiro et al. 2016). Herbs such as *Piper methysticum* (kava kava), and *Ziziphus jujuba*’s dried seed (suanzaoren) are widely used to treat sleep issues, stress, and anxiety (Cho and Shimizu 2015; Shi et al. 2014; Yang et al. 2023). Natural supplements like melatonin are used to treat shift work‐related insomnia and delayed sleep phase syndrome, with vitamins and minerals also shown to benefit those with specific sleep disorders (Meletis and Zabriskie 2008). In addition, a number of natural compounds include antioxidant, neuroprotective, and anti‐inflammatory properties that enhance brain function in general and alleviate insomnia and stress (Rehman et al. 2019; Al Hasan et al. 2025).

L‐Quebrachitol (LQL) (C_7_H_14_O_6_) is also known as quebrachitol, which is found in nature, and it is an optically active cyclitol (Liang et al. 2020). LQL is obtained from natural plants such as *Hevea brasiliensis*, *Allophylus edulis*, and *Cannabis sativa* (Hu et al. 2021; dos Santos et al. 2023; Ehigiator et al. 2021). LQL has a large number of biological activities, including anticancer activity (Rattajak et al. 2024), antidiabetic activity (Mu et al. 2022), antimicrobial activity (Vijayakumar et al. 2020). A study found that LQL extracted from industrial rubber serum exhibits potent antioxidant activities (Li et al. 2023) and enhances bone mineral density (Li et al. 2023). Another research again showed that LQL enhances the proliferation, differentiation, and mineralization of MC3T3‐E1 cells through the activation of the BMP‐2/Runx2/MAPK/Wnt/β‐catenin signaling pathway (Yodthong et al. 2018). On the other hand, LQL treatment improved glucose uptake, reduced lipid accumulation, and modulated key metabolic gene expressions in insulin‐resistant HepG2 cells (Mu et al. 2022). LQL shares close structural similarity with myo‐inositol (Huang et al. 2025), a compound known to regulate neuronal signaling and GABAergic neurotransmission (Elhadidy and Salama 2025). This structural resemblance suggests that LQL may also interact with the gamma‐aminobutyric acid (GABA) receptor system, which plays a critical role in sedation and anxiety control. Moreover, several studies have reported that LQL exhibits strong antioxidant, anti‐inflammatory, and neuroprotective effects, which are often associated with improved CNS function and resistance to stress‐related disorders (Li et al. 2023; Asrafi et al. 2025). Although no clinical studies have yet evaluated its sedative efficacy, its favorable safety profile, low toxicity (*LD*_50_ = 804 mg/kg), and pharmacokinetic properties support its potential translational relevance. Based on these findings, LQL was selected as a promising candidate for investigating sedative effects through GABAergic mechanisms.

For this, our study aimed to investigate the sedative effect of LQL using a TS‐induced sleeping protocol in chicks and performed in silico studies with GABA_A_ receptor‐responsible subunits.

## 2 Martials and Methods

### 2.1 In Vivo Study

#### 2.1.1 Chemicals and Reagents

LQL (CAS: 642‐38‐6, purity: 98% [HPLC]) was bought from Chengdu Alfa Biotechnology, China, while the standard drug diazepam (DZP) and the inducer TS were kindly provided by Square Pharmaceuticals Ltd., Bangladesh. Tween 80 and NaCl required for this study were purchased from Merck (India).

#### 2.1.2 Experimental Animals

Young broiler chicks (*Gallus gallus domesticus*) of either sex, with a body weight range of 38–45 g, 2 days old, were purchased from a local market in Khulna, Bangladesh. The chicks were acclimatized for 2 days at the pharmacology lab of Gopalganj Science and Technology University (GSTU) before starting this study. During this time, the chicks had free access to standard foods and water ad libitum. Room temperature was maintained at 27 ± 2°C with a 12‐h dark/light cycle under controlled illumination. After 12 h of fasting, this study was carried out. However, they were allowed free access to water only. Studies were performed between 9:00 a.m. and 3:00 p.m. This study was approved by the Animal Ethics Committee of Khulna University (KUAEC‐2023‐05‐09).

#### 2.1.3 Selection and Administration of Doses

In this study, chicks were administered LQL at doses of 1, 5, and 10 mg/kg. These dose levels were chosen based on the findings of a previous study conducted by Asrafi et al. (2025) (Asrafi et al. 2025). Before the experiment, all animals were fasted overnight. The chicks were randomly categorized into different groups, each comprising five animals. All the treatments were given 30 min before TS administration at 10 mg/kg. TS and all the treatments, including combination therapy, were given via the ip route. Then follow the below‐mentioned study.

#### 2.1.4 Sedative Effect Study in Broiler Chicks

##### 2.1.4.1 Study Design

In this study, a total of 30 chicks were randomly divided into six groups, each containing five animals (*n* = 5). The number of animals per group (*n* = 5) was determined based on previous experimental reports evaluating the sedative effects of natural compounds using TS‐induced sleep models in chicks and mice (Al Hasan et al. 2025). Both male and female healthy chicks were evenly distributed among the groups to avoid sex bias. Each chick was marked with a unique identification code to ensure unbiased allocation. Data collection and behavioral scoring were conducted by an investigator blinded to the treatment conditions to minimize experimental and observer bias. Treatment groups and their details have been shown in Table 1.

**TABLE 1:** Treatment groups with their details at 10 mL/kg volume of administration.

Treatment groups	Description	Administration design
Individual treatment groups		
Control vehicle	Distilled water containing 0.9% NaCl and 0.5% tween 80	ip, at a time
DZP‐2	Diazepam (GABA receptor agonist reference drug) at 2 mg/kg	ip, at a time
LQL‐1	L‐quebrachitol (test sample) at 1 mg/kg	ip, at a time
LQL‐5	L‐quebrachitol (test sample) at 5 mg/kg	ip, at a time
LQL‐10	L‐quebrachitol (test sample) at 10 mg/kg	ip, at a time
Combination treatment group		
LQL‐10+DZP‐2	L‐quebrachitol l0 mg/kg + diazepam 2 mg/kg	ip + ip, one followed by another

*Note*: At a time, drug was given in a single dose.

Abbreviations: DZP, diazepam; ip, intraperitoneal (*n *= 5); LQL, L‐quebrachitol.

##### 2.1.4.2 TS‐Induced Sleeping Study in Chicks

This study was carried out according to the method described by Hassan et al. (2024). The test sample (LQL), control (vehicle: 10 mL/kg), and the GABA receptor agonist drug DZP (2 mg/kg) were administered via the ip route. After 30 min, sleep inducer TS (10 mg/kg, ip) was administered to each chick, and it was observed for the latency (time between TS administration and appearance of sleep) and sleep duration (total sleep time).

#### 2.1.5 Statistical Analysis

Values are expressed as the mean ± SEM (standard error of the mean). One‐way analysis of variance (ANOVA) followed by a Tukey multiple comparison test at 95% confidence intervals using GraphPad Prism software (version 9.5, San Diego, USA). Data were considered significant when *p < *0.05.

### 2.2 In Silico Studies

#### 2.2.1 Ligand Collection and Preparation

**FIGURE 1:**
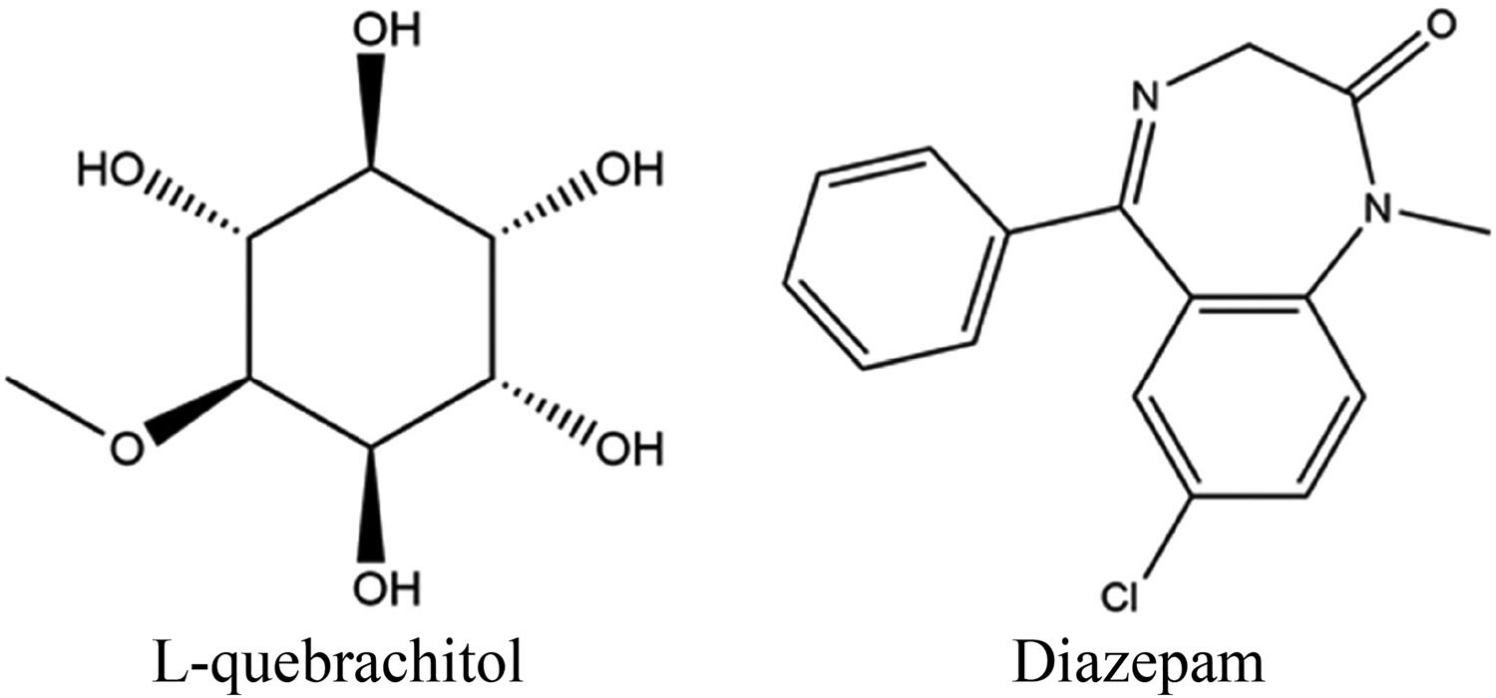
The 2D chemical structures of LQL and diazepam.

The three‐dimensional structures of the selected ligands were acquired from the PubChem online database (https://pubchem.ncbi.nlm.nih.gov/) to prepare for the docking investigations. These included DZP (PubChem ID: 3016) and LQL (PubChem ID: 151108). The 3D structures were downloaded in SDF format. We optimized the ligands by reducing energy using the MM2 (Allinger's force field) method using Chem3D Pro 20.1.1 176 software (Islam et al. 2024a; Islam et al. 2024e). Figure 1 depicts the 2D structure of LQL and DZP.

#### 2.2.2 Protein Collection and Preparation

We have chosen the GABA_A_ receptor subunits α1 and β2, which are the sedation‐involving receptors. The RCSB Protein Data Bank (https://www.rcsb.org/), which was retrieved on October 6, 2024, provided the three‐dimensional structures of the GABA_A_ receptor (PDB ID: 6X3X) (https://www.rcsb.org/structure/6x3X). PyMol (v2.4.1) software has been used to eliminate any unnecessary components from the protein structure, such as chains, heteroatoms, and water molecules, in order to optimize receptor function and avoid docking disruption. The SwissPDB Viewer program was used to optimize the receptor design in order to carry out the molecular docking procedure while conserving energy. Optimization in Swiss PDBViewer involves loading the structure, adding hydrogens, performing energy minimization using the GROMOS96 force field, refining ligand conformation, optimizing side‐chain rotamers, and saving the refined structure (Islam et al. 2024b; Bhuia et al. 2023; Husain et al. 2024).

#### 2.2.3 Molecular Docking Study

To determine the binding energy of the selected ligands against the GABA_A_ receptor (PDB: 6X3X), we used PyRx v0.8. The dimensions of the grid box were 20.01 × 18.54 × 17.29 A° for the *x*, *y*, and *z* axes, respectively. The computation was supported by a 200‐step calculation, and the docking result was then saved in a “csv” format. Using the programs PyMol (v2.4.1) and Discovery Studio (v21.1.020298), the ligand‐protein configuration has been shown (Islam et al. 2024c; Chowdhury et al. 2023; Al Hasan, Mia, et al. 2024).

#### 2.2.4 Prediction of Pharmacokinetics and Drug‐Likeness

Pharmacokinetics predictions and drug‐likeness occur to determine the absorption, distribution, metabolism, excretion, and toxicity (ADMET) properties of the compounds (Islam et al. 2024d). The SwissADME (http://www.swissadme.ch/) and pkCSM (https://biosig.lab.uq.edu.au/pkcsm/) online tool has been employed to estimate the pharmacokinetics and physicochemical properties of LQL.

#### 2.2.5 Toxicity Prediction

The toxicity profile of LQL was evaluated utilizing the ProTox 3.0 web server (https://tox.charite.de/protox3/), which was primarily utilized to investigate parameters such as hepatotoxicity, neurotoxicity, immunotoxicity, mutagenicity, cardiotoxicity, carcinogenicity, clinical toxicity, and cytotoxicity.

## 3 Results

### 3.1 In Vivo Findings

According to Table 2, the in vivo experiment exhibited that the standard drug (DZP‐2) significantly (*p < *0.05) decreased the latency period (60.00 ± 5.91 s) in treated chicks compared to the control group (132.00 ± 2.00 s). LQL‐1 significantly (*p* < 0.05) reduced the latency period (114.20 ± 5.05 s) in contrast to the animals of the control group. Further, LQL‐5 significantly (*p < *0.05) diminished the latency period (98.00 ± 3.59 s) in comparison with the control group. Furthermore, when administered, LQL‐10 significantly (*p < *0.05) reduced the latency (56.00 ± 6.77 s) compared to the control and DZP‐2 groups in a dose‐dependent manner. When DZP‐2 combined with LQL‐10 revealed the lowest latency period (45.80 ± 4.50 s) in contrast with their individual and the control groups.

**TABLE 2:** Latency and sleeping time observed in different treatment groups of animals.

Treatment groups	Latency (s)	Sleep duration (min)
Control^*^	132.00 ± 2.00	59.20 ± 7.30
DZP‐2^a^	60.00 ± 5.91^*^ ^b^, ^c^	75.00 ± 6.86^*^
LQL‐1^b^	114.20 ± 5.05^*^	70.80 ± 4.33^*^
LQL‐5^c^	98.00 ± 3.59^*^ ^b^	81.00 ± 7.18^*^ ^a^, ^b^
LQL‐10^d^	56.00 ± 6.77^*^ ^a^, ^b^, ^c^	103.20 ± 3.55^*^ ^a^, ^b^, ^c^
LQL‐10+DZP‐2^e^	45.80 ± 4.50^*^ ^a^, ^b^, ^c^, ^d^	115.60 ± 5.23^*^ ^a^, ^b^, ^c^, ^d^

*Note*: Values are mean ± SEM (*n* = 5), One‐way ANOVA followed by Tukey multiple comparison test, *p* < 0.05 compared to the *Control (vehicle: distilled water containing 0.9% NaCl and 0.5% tween 80).

^a^ DZP‐2 (diazepam at 2 mg/kg).

^b^ LQL‐1 (L‐quebrachitol at 1 mg/kg) group.

^c^ LQL‐5 (L‐quebrachitol at 5 mg/kg) group.

^d^ LQL‐10 (L‐quebrachitol at 10 mg/kg) group.

^e^ LQL‐10+DZP‐2 (L‐quebrachitol at 10 mg/kg + diazepam at 2 mg/kg).

In this investigation, DZP‐2 significantly (*p < *0.05) increased the sleeping duration (75.00 ± 6.86 min) in comparison with the control (59.20 ± 7.30 min). The test sample (LQL‐1) also significantly (*p* < 0.05) enhanced sleep duration (70.80 ± 4.33 min) compared to the control group. When administered, LQL‐5 and 10 significantly (*p < *0.05) elevated the sleeping duration (81.00 ± 7.18 and 103.20 ± 3.55 min, respectively) in contrast with DZP‐2 and the control groups in a dose‐dependent manner. In combination therapy, when administered, DZP‐2, along with LQL‐10, significantly (*p < *0.05) displayed the maximum sleeping duration (115.60 ± 5.23 min) relative to the other treatment group. The latency period and sleeping duration of the test and standard drug groups are represented in Table 2.

### 3.2 In Silico Findings

**FIGURE 2:**
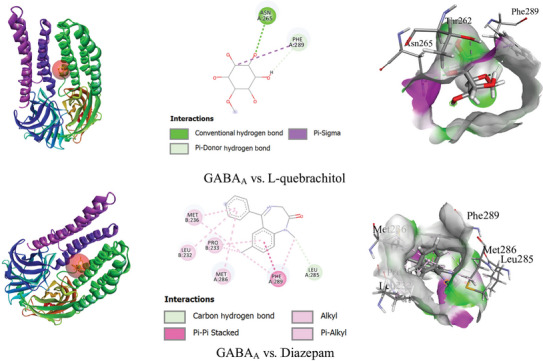
The 2D and 3D non‐bond interaction of L‐quebrachitol and diazepam with the GABA_A_ receptor (α1 and β2) (6X3X).

In silico investigation revealed that DZP exhibited a higher score (−8.3 kcal/mol) towards GABA_A_ receptors than LQL (−5.3 kcal/mol). LQL formed two hydrogen bonds (HBs) with ASN A: 265 (2.32 Å) and PHE A: 289 (2.79 Å) amino acid (AA) residues as well as a hydrophobic (HP) bond with AA residues such as PHE A: 289. Whereas, DZP demonstrated only one HB with LEU A: 285 (3.65 Å) and some HP bonds with AA residues, including PHE A: 289, PRO B: 233, LEU A: 285, MET B: 236, MET A: 286, and LEU B: 232. Table 3 shows the docking score, bond types, number of HBs, HB lengths, and list of AA residues that interact with ligands. Figure 2 demonstrates the 2D and 3D non‐bond interactions of LQL and DZP with the GABA_A_ receptor (α1 and β2) (6X3X).

**TABLE 3:** Molecular docking scores of L‐quebrachitol and diazepam against GABA_A_ (α1 and β2 subunits) receptor.

Ligand‐receptor	Binding score (kcal/mol)	Amino acid residues	
		Hydrogen bond with length (Å)	Other bonds
LQL−GABA_A_ (α1 and β2 subunits)	−5.3	ASN A:265 (2.32) PHE A:289 (2.79)	PHE A: 289
DZP−GABA_A_ (α1 and β2 subunits)	−8.3	LEU A:285 (3.65)	PHE A: 289 PRO B: 233 LEU A: 285 MET B: 236 MET A: 286 LEU B: 232

### 3.3 Estimation of Drug‐Likeness and Pharmacokinetics

**FIGURE 3:**
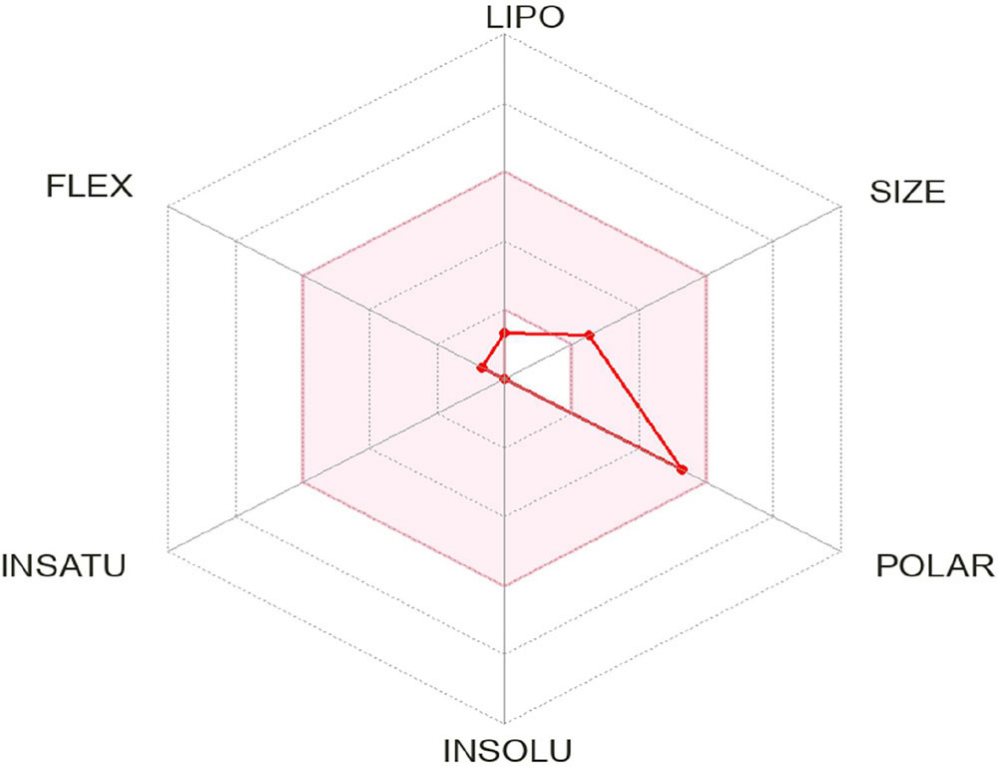
The physicochemical properties of L‐quebrachitol. (The colored zone is the suitable physicochemical space for oral bioavailability; LIPO [lipophilicity]: −0.7 < XLOGP3 < +5.0; SIZE: 150 g/mol < molecular weight [MW] < 500 g/mol; POLAR [polarity]: 20 Å2 < topological polar surface area [TPSA] < 130 Å2 ; INSOLU [insolubility]: −6 < log *S* [ESOL] < 0; INSATU [in saturation]: 0.15 < Fraction Csp 3 < 1; FLEX [flexibility]: 0 ≤ number of rotatable bonds < 9).

In this pharmacokinetics prediction, LQL has a molecular weight of 194.18 g/mol, featuring five hydrogen bond donors (HBD) and six hydrogen bond acceptors (HBA). Its negative log *P*_o/w_ value of −3.17 indicates strong water solubility, and its log *S* (ESOL) value of 1.02 further supports this highly solubility in water. The molecule adheres to Lipinski's Rule of five with moderate oral bioavailability (0.55) and limited lipophilicity. However, it exhibited poor permeability across the blood‐brain barrier (log *BB*: −1.053) and low CNS penetration (log *PS*: −4.019). In addition, it does not interact with P‐glycoproteins or major CYP enzymes. However, all parameters involved in these criteria are shown in Table 4 and Figure 3.

**TABLE 4:** Pharmacokinetic properties of L‐quebrachitol predicted by SwissADME and pkCSM online server.

Properties	Parameters	L‐quebrachitol
Physicochemical properties	Formula	C_7_H_14_O_6_
	MW (g/mol)	194.18
	No. of heavy atoms	13
	No. of aromatic heavy atoms	0
	No. of HBD	5
	No. of HBA	6
	MR	40.54
Lipophilicity	Log *P*_o/w_ (XLOGP3)	−3.17
Drug‐likeness	Lipinski	Yes
	Bioavailability score	0.55
Water solubility	Log *S* (ESOL)	1.02
	Class	Highly soluble
Absorption	Caco‐2 permeability (log *Papp* in 10^−6^ cm/s)	−0.14
	Intestinal absorption (human) numeric (% absorbed)	36.202
	Skin permeability (log *Kp* cm/h)	−2.997
	P‐glycoprotein I inhibitor	No
	P‐glycoprotein II inhibitor	No
Distribution	BBB permeability (log *BB*)	−1.053
	CNS permeability (log *PS*)	−4.019
	VDss (human) (log L/kg)	−4.019
Metabolism	CYP2D6 substrate	No
	CYP3A4 substrate	No
	CYP2D6 inhibitor	No
	CYP3A4 inhibitor	No
Excretion	Total clearance (log mL/min/kg)	0.659
	Renal OCT2 substrate	No

Abbreviations: BBB, blood‐brain barrier; CNS, central nervous system; CYP, cytochrome P450; HBA, hydrogen bond acceptors; HBD, hydrogen bond donors; LD50, median lethal dose; MR, molar refractivity; MW, molecular weight; VDss, volume of distribution (at steady state).

### 3.4 Assessment of Toxicity Predictions of the Selected Compounds

In our toxicity prediction, LQL showed a toxicity class of four with an LD_50_ of 804 mg/kg. No toxicity involving hepatotoxicity, neurotoxicity, immunotoxicity, carcinogenicity, clinical toxicity, or cytotoxicity was found (Table 5).

**TABLE 5:** The toxicity profile of L‐quebrachitol with different parameters predicted by the ProTox 3.0 server.

Properties	Parameters	L‐quebrachitol
Toxicity	TC	4
	Predicted LD_50_ (mg/kg)	804
	Hepatotoxicity	Inactive
	Neurotoxicity	Inactive
	Immunotoxicity	Inactive
	Mutagenicity	Inactive
	Carcinogenicity	Inactive
	Clinical toxicity	Inactive
	Cytotoxicity	Inactive
	Ecotoxicity	Inactive

Abbreviations: LD_50_, median lethal dose; TC, toxicity class.

## 4 Discussion

Sedation is mainly mediated through the activation of GABA receptors, particularly the GABA_A_ receptors, which enhance the inhibitory neurotransmission in the CNS (Philip et al. 2025). When GABA binds to the GABA_A_ receptors, the chloride channel in the postsynaptic neuron opens, allowing the chloride ion to enter the cell. Due to the influx of chloride ions, hyperpolarization occurs in the postsynaptic membrane (Lombardi et al. 2021). GABA plays a key role in reducing neuronal excitability by keeping channels open longer (Kihara 2024), while pharmacological agents like benzodiazepines and barbiturates are used for their CNS depressant, muscle relaxation, and anxiolytic properties (Griffin et al. 2013).

**FIGURE 4:**
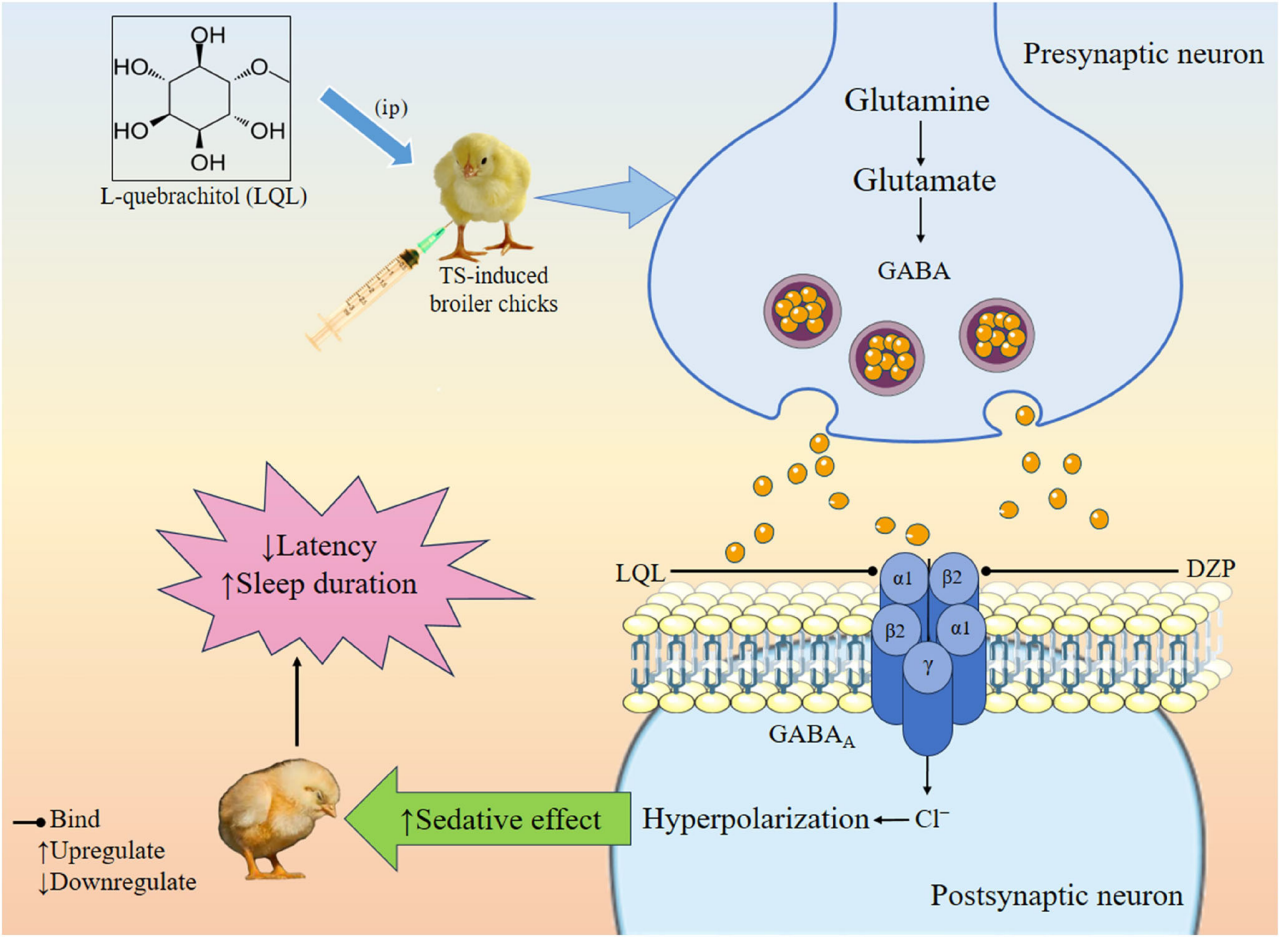
The possible sedative mechanism of L‐quebrachitol. This diagram illustrates the sedative effects of LQL on TS‐induced broiler chicks. It shows the administration of LQL (ip), which influences GABAergic signaling by interacting with GABA_A_ receptors, leading to increased chloride ion (Cl^−^) influx and hyperpolarization of postsynaptic neurons. The result is decreased sleep latency and prolonged sleep duration. LQL's action is compared with DZP in enhancing sedative effects. By enhancing GABA's inhibitory function, LQL significantly boosts the calming and sedative effects on the nervous system. The process demonstrates how LQL may alter neuronal activity, contributing to its sedative effects. DZP, diazepam; GABA_A_, gamma‐aminobutyric acid type A; LQL, L‐quebrachitol.

Barbiturates are a class of sedative‐hypnotic drugs that act on the GABA_A_ receptors and exert their action by increasing the duration of receptor channel opening (Richardson et al. 2024). TS, a barbiturate, exerts sedative activity by modulating GABAA receptors, enhancing chloride ion channel opening, and leading to hyperpolarization of the postsynaptic neuron, which reduces neuronal activity (Al Hasan, Bhuia, et al. 2024). TS‐induced sleeping protocol is a common method to assess the sedative effects of test compounds, focusing on their interaction with the GABAergic pathway by measuring sleep latency, duration, and quality (Bappi et al. 2023). TS modulates GABA receptor activity, enhancing chloride influx, hyperpolarizing neurons, and promoting sedation (de la Peña et al. 2013). Standard tools, such as electroencephalograms (EEG), are often employed to monitor sleep stages and patterns (Pile et al. 2022). In our behavioral experiment, the powerful sedative effects of DZP are clearly evident. DZP significantly (*p* < 0.05) reduced the sleep latency and noticeably extended the overall sleep duration. Compared to the control group, our test compound LQL reduced latency and extended the duration of sleep in a dosage‐dependent manner. In addition, at the higher dose (LQL‐10), it showed a more enhanced effect than DZP. Moreover, the combination of DZP and LQL‐10 resulted in the best observation on sleep duration and latency, where the combination therapy significantly (*p* < 0.05) demonstrated the lowest sleep latency with a maximum sleep duration. These findings in the combination therapy indicate a better sedative action. DZP is a well‐known sedative acting via GABA_A_ receptor modulation. Previous studies, such as Al Hasan et al. (2025), reported that natural compounds like tangeretin enhance DZP's sedative efficacy (Al Hasan et al. 2025). Based on this, our study examined the combination of LQL with DZP, revealing enhanced sedation compared to either treatment alone. However, further dose optimization is needed. Future studies should evaluate higher LQL doses (> 10 mg/kg) without DZP to establish the dose–response relationship, determine the maximal effective dose, and assess safety. This will clarify LQL's intrinsic sedative potential and its suitability as an adjunct to conventional sedatives. However, the possible sedative mechanism of LQL is shown in Figure 4.

Molecular docking is a computational technique used to predict the interaction between a ligand and a target macromolecule, such as a protein or nucleic acid (Pinzi and Rastelli 2019). Molecular docking is a vital tool in drug discovery and structural biology, aiding in the identification of therapeutic compounds by simulating BA activity and optimizing lead compounds for further studies (Meng et al. 2011; Pagadala et al. 2017). In our study, LQL demonstrates a moderate BA (−5.3 kcal/mol) towards GABA_A_ receptors, indicating LQL may show a lesser extent of sedative effects than DZP. In drug discovery, similar AA residues help optimize target binding, specificity, and selectivity in drug design, improving efficacy and minimizing resistance (Al Hasan, Bhuia, et al. 2024). Both LQL and DZP have an interesting interaction with the GABA_A_ receptor, particularly with the AA residue PHE A: 289. This similar interaction site indicates that LQL might also have potential therapeutic effects related to the GABA_A_ receptor, like DZP. HBs are crucial for stabilizing ligand‐receptor complexes, enhancing binding specificity, and ensuring precise molecular recognition through significant interaction strength (Du et al. 2023). In silico findings also revealed that LQL formed two HBs with GABA_A_ receptors, specifically with ASN A: 265 (2.32 Å) and PHE A: 289 (2.79 Å). In contrast, DZP demonstrated only one HB with LEU A: 285 (3.65 Å). This indicates that LQL exhibits a more extensive HB interaction compared to DZP, potentially contributing to its binding specificity. The stronger HB interaction and similar AA residues of LQL with GABA_A_ receptors suggest its potential as a promising drug candidate for targeting these receptors.

Pharmacokinetics is the study of how medications are absorbed, distributed, metabolized, and excreted, and how they interact with the body (Alagga et al. 2024). Solubility and cellular permeability are crucial biopharmaceutical characteristics in drug development, as low solubility compounds complicate both in vitro and in vivo testing (Burton and Goodwin 2010; Di et al. 2012). Our study suggests that LQL, with a molecular weight of 194.18 g/mol, is highly hydrophilic and water‐soluble (log *S* 1.02). Lipinski's rule of five demonstrates the bioavailability of a compound (Ivanović et al. 2020), indicating good drug‐likeness with low lipophilicity (log *P*_o/w_ −3.17) and moderate bioavailability (0.55). Despite moderate absorption (36.2%), its poor blood‐brain barrier permeability limits CNS activity. The compound's lack of interaction with key CYP enzymes or P‐glycoproteins suggests minimal interference with drug metabolism or transport, making it a promising candidate with specific pharmacokinetic characteristics.

Although no experimental pharmacokinetic or pharmacodynamic data on LQL are currently available, the in silico predictions presented in this study provide valuable preliminary insights into its biopharmaceutical characteristics. The molecular docking outcomes suggest that LQL may interact with GABA_A_ receptor subunits (α_1_ and β_2_), indicating a possible GABAergic mechanism underlying its sedative potential. Future research should focus on conducting comprehensive in vivo pharmacokinetic and pharmacodynamic studies to validate these predictions, clarify its ADME properties, and establish dose–response relationships.

Toxicity prediction is the process of using computational models to assess the potential harmful effects of substances on biological systems (Pérez Santín et al. 2021). LQL is classified as having low toxicity (toxicity Class 4) with an LD_50_ of 804 mg/kg. It exhibits no adverse effects related to several toxicities, which suggests that LQL is relatively safe for biological systems at exposure levels.

Our study highlights that LQL exhibited dose‐dependent sedative effects, with its higher dose (LQL‐10) showing almost similar efficacy as DZP and better action in combination therapy with DZP. Molecular docking revealed LQL interacts moderately with GABA_A_ receptors (α1 and β2 subunits) (−5.3 kcal/mol), forming strong HB interactions and similar AA residues with standard drug DZP, suggesting potential therapeutic effects. LQL also demonstrated favorable drug‐likeness with low toxicity. Despite moderate absorption, its safety and pharmacokinetic profile make it a promising drug candidate. These findings underscore LQL's potential for development as a sedative agent targeting GABA_A_ receptors (α1 and β2 subunits).

While this study provides valuable insights into the sedative potential of LQL, several limitations should be acknowledged. The present findings are primarily based on preliminary in vivo and in silico analyses, which, although informative, require further validation. The study lacks in vitro receptor‐binding or electrophysiological assays to confirm LQL's affinity and mechanism of interaction with GABA_A_ receptor subunits. Moreover, assessments of biological membrane permeability and blood‐brain barrier penetration were limited to computational predictions and not experimentally verified. The animal sample size was relatively small, and only one species and model were used, which may restrict the generalizability of the findings. Furthermore, no pharmacokinetic or toxicokinetic studies were conducted in vivo to support the computational safety predictions. Therefore, future research should focus on detailed in vitro and in vivo investigations to elucidate the molecular mechanisms, pharmacokinetic behavior, and bioavailability of LQL. Ultimately, well‐designed clinical trials will be necessary to evaluate its safety, optimal dosing, and therapeutic efficacy in humans.

## 5 Conclusion

In summary, the present study demonstrates that LQL exerts significant sedative effects in a dose‐dependent manner in thiopental sodium‐induced chicks. The combination of LQL (10 mg/kg) with DZP (2 mg/kg) produced enhanced sedative activity compared to individual treatments, suggesting a synergistic action through modulation of the GABAergic system. Molecular docking results further supported its potential interaction with GABA_A_ receptor subunits (α_1_ and β_2_), indicating a plausible mechanism of action. In silico pharmacokinetic and toxicity predictions revealed favorable solubility, stability, and safety profiles, supporting its potential as a safe sedative candidate. Overall, LQL represents a promising natural compound for sedative drug development, though future in vitro, pharmacokinetic, and clinical studies are necessary to validate its efficacy, mechanism, and therapeutic safety.

## Author Contributions

All authors made a significant contribution to the work reported, whether that is in the conception, study design, execution, acquisition of data, analysis, and interpretation, or in all these areas that is, revising or critically reviewing the article; giving final approval of the version to be published; agreeing on the journal to which the article has been submitted; and confirming to be accountable for all aspects of the work. All authors have read and agreed to the published version of the manuscript.

## Funding

This study was supported by the University Higher Education Fund number CL/CO/A/8.

## Ethics Statement

This study was approved by the Animal Ethics Committee of Khulna University (KUAEC‐2023‐05‐09).

## Conflicts of Interest

The authors declare no conflicts of interest.

## Data Availability

Data will be made available on request.
